# Cryo-EM reveals the asymmetric assembly of squid hemocyanin

**DOI:** 10.1107/S205225251900321X

**Published:** 2019-04-05

**Authors:** Yoshikazu Tanaka, Sanae Kato, Markus Stabrin, Stefan Raunser, Takashi Matsui, Christos Gatsogiannis

**Affiliations:** a Graduate School of Life Sciences, Tohoku University, 2-1-1 Katahira, Aoba-ku, Sendai 980-8577, Japan; b Japan Science and Technology Agency, PRESTO, 2-1-1 Katahira, Aoba-ku, Sendai 980-8577, Japan; c Faculty of Fisheries, Kagoshima University, Kagoshima 890-0056, Japan; d The United Graduate School of Agricultural Sciences, Kagoshima University, Kagoshima 890-0056, Japan; e Max Planck Institute of Molecular Physiology, Department of Structural Biochemistry, Otto Hahn Strasse 11, Dortmund 44227, Germany

**Keywords:** single-particle cryo-EM, structure determination, cryo-electron microscopy, macromolecular machines, protein structures

## Abstract

The oxygen transporter of molluscs, hemocyanin, consists of long pearl-necklace-like subunits of several globular domains. The subunits assemble in a complex manner to form cylindrical decamers. Typically, the first six domains of each subunit assemble together to form the cylinder wall, while the C-terminal domains form a collar that fills or caps the cylinder. During evolution, various molluscs have been able to fine-tune their oxygen binding by deleting or adding C-terminal domains and adjusting their inner-collar architecture. However, squids have duplicated one of the wall domains of their subunits instead. Here, using cryo-EM and an optimized refinement protocol implemented in *SPHIRE*, this work tackled the symmetry-mismatched structure of squid hemocyanin, revealing the precise effect of this duplication on its quaternary structure and providing a potential model for its structural evolution.

## Abstract 2

The cryo-EM structure of squid hemocyanin, obtained using a multi-symmetry refinement protocol in *SPHIRE*, reveals an unexpected breaking of fivefold symmetry in the central ring and a striking arrangement of the subunits.

## 1. Introduction   

Oxygen transportation is one of the most important events for living organisms. Some animals such as molluscs and arthropods have blue blood because they utilize hemocyanin, a type-3 copper-containing protein that freely dissolves in hemolymph for oxygen transportation (Decker & Terwilliger, 2000 ▸; Markl, 2013 ▸; van Holde *et al.*, 2001 ▸).

Molluscan hemocyanins form decamers or multidecamers of 330–550 kDa subunits, which associate into huge cylindrical supermolecules with molecular masses varying from 3.5 to 13.5 MDa. Therefore, molluscan hemocyanins are acknowledged to be among the largest known protein complexes (Markl, 2013 ▸). They have bioengineering applications, *e.g.* as adjuvants for antibody preparation and carrier molecules for vaccines (Becker *et al.*, 2014 ▸), because of their enormous size and the presence of the carbohydrate modifications on their surface (Geyer *et al.*, 2005 ▸; Harris & Markl, 1999 ▸; Siddiqui *et al.*, 2007 ▸).

Subunits of most molluscan hemocyanins consist of an N-terminal segment of six paralogous functional units (FUs; a-b-c-d-e-f) and a C-terminal segment with a varying number of FUs (Kato *et al.*, 2018 ▸; Lieb & Markl, 2004 ▸; Markl, 2013 ▸). Each FU contains a single oxygen binding site and up to three binding sites for carbohydrates. The paralogous FUs are connected by short flexible linker peptides, and the resulting subunits associate to form cylindrical decamers. Ten copies of the N-terminal segment (FUs a, b, c, d, e and f) thereby form a conserved cylinder wall, whereas the C-terminal segments form several inner-collar domains, located on the inner periphery of the cylinder. The structure of molluscan hemocyanins has been extensively studied for several decades mainly by the combination of single-particle electron cryo-microscopy (cryo-EM) and X-ray crystallography (Cuff *et al.*, 1998 ▸; Gai *et al.*, 2015 ▸; Gatsogiannis & Markl, 2009 ▸; Gatsogiannis *et al.*, 2007 ▸, 2015 ▸; Jaenicke *et al.*, 2010 ▸; Perbandt *et al.*, 2003 ▸; Zhang *et al.*, 2013 ▸; Zhu *et al.*, 2014 ▸).

Depending on the subunit composition and overall architecture, molluscan hemocyanins can be classified into the following types: (1) keyhole limpet type (a-b-c-d-e-f-g-h), (2) mega-hemocyanin type (a-b-c-d-e-f-f1-f2-f3-f4-f5-f6), (3) nautilus type (a-b-c-d-e-f-g) and (4) squid type (a-b-c-d-d*-e-f-g) (Fig. S1 of the supporting information).

The architecture of the inner-collar domains differs depending on the FU composition of the respective C-terminal segments. Furthermore, according to the differences in the structure of the inner collar, there is an impact on the architecture of the entire molecule, *i.e.* with regard to the capability to form decamers, di-decamers and multi-decamers.

This wealth of data has provided a deeper understanding of the structure and evolution of hemocyanin and revealed the plasticity of the inner-collar architecture (addition or removal of C-terminal FUs) as a possible molecular tool to optimize oxygen binding according to the environmental conditions and physiology of the respective molluscan class. Therefore, structural studies of hemocyanins have been of significant importance from the viewpoint of structural protein evolution (Decker *et al.*, 2007 ▸; Markl, 2013 ▸; Thonig *et al.*, 2014 ▸).

Type 4 molluscan hemocyanin (squid type) is exceptional since this is the only molluscan hemocyanin type with an additional FU (FU-d*) within the N-terminal segment that usually forms the cylinder wall (Boisset & Mouche, 2000 ▸; Gai *et al.*, 2015 ▸; Lambert *et al.*, 1995 ▸). Structural studies of this particular hemocyanin type are crucial to unveiling the effect of this exceptional gene duplication on the overall arrangement of the decamer. This type of hemocyanin was only recently analyzed by X-ray crystallography, *e.g.* a structure of *Todarodes pacificus* hemocyanin (TpH), a squid-type 3.5 MDa hemocyanin composed of FUs-a-b-c-d-d*-e-f-g, was obtained at a 3.0 Å resolution (Gai *et al.*, 2015 ▸).

The X-ray structure showed a typical decameric hollow cylindrical wall with *D*_5_ symmetry; however, the inner-collar domains were not resolved and the *D*_5_ symmetry of the inner-collar structure was apparently incorrect. Consequently, the precise architecture of the inner collar and topology of FU-d* and FU-g still remain unclear.

Here we applied cryo-EM and an optimized protocol for single-particle analysis of symmetry-mismatched complexes using *SPHIRE* (Moriya *et al.*, 2017 ▸) in order to reveal the structure of the inner-collar domains of type 4 hemocyanin and complete the picture of the structural evolution of molluscan hemocyanin.

## 2. Results and discussion   

### 2.1. Cryo-EM analysis of TpH   

**Figure 1:**
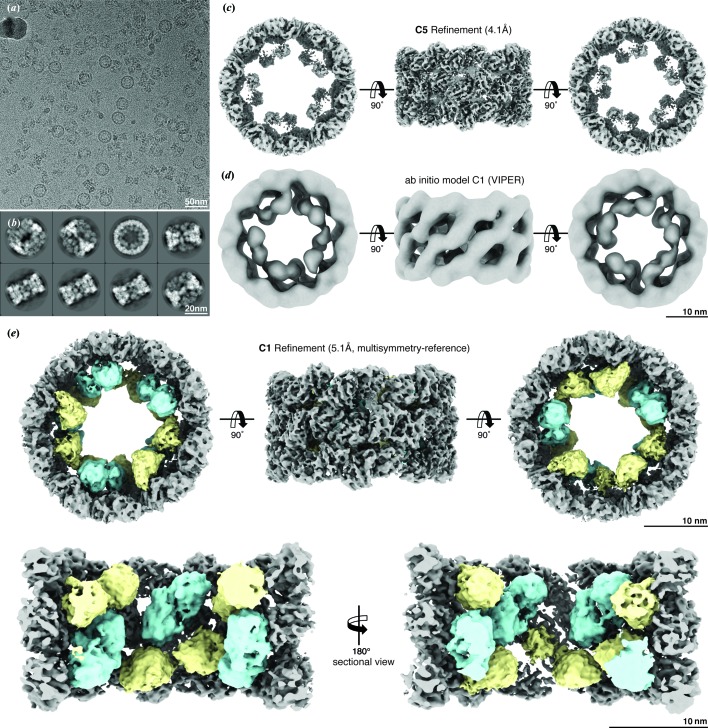
Cryo-EM density of TpH. (*a*) Representative cryo-EM micrograph of vitrified TpH (scale bar: 50 nm). (*b*) Representative class averages (scale bar: 20 nm). (*c*) Cryo-EM structure of TpH refined with *C*_5_ symmetry imposed. (*d*) The *ab initio* model calculated using *RVIPER* with no symmetry imposed. (*e*) Final cryo-EM structure of TpH refined with no symmetry imposed. The wall region, FU-gs and FU-d*s are shown in gray, cyan and yellow, respectively. Cut-open views are also shown.

We purified hemocyanin from the hemolymph of the Japanese flying squid and used cryo-EM with direct electron detection and single-particle analysis to determine its structure [Fig. 1 ▸(*a*)]. *SPHIRE* was used for all image-processing steps. A total of 359 250 particles were subjected to 2D classification using ISAC (Yang *et al.*, 2012 ▸) and a subset of 196 315 particles were identified that could form stable and reproducible 2D class averages [Fig. 1 ▸(*b*)]. This subset of particles was used for further analysis.

Previous low-resolution negative-stain studies of this hemocyanin type suggested *D*_5_ symmetry for the entire complex (Lambert *et al.*, 1995 ▸; Mouche *et al.*, 1999 ▸). The previous crystal structure of TpH showed an overall *D*_5_ symmetry, but the inner-collar architecture and the respective domains could not be resolved (Gai *et al.*, 2015 ▸). The rather ‘artificial’ *D*_5_ symmetry of the inner collar was explained as the result of crystal packing mediated by face-to-face interactions of two vertically opposite decamers with *C*_5_ symmetry. Taking into account that, at first glance, the top-view ISAC class averages also indicated cyclic fivefold symmetry for the inner collar [Fig. 1 ▸(*b*)], we used a typical cylinder wall as the reference and routinely imposed *C*_5_ symmetry during the refinement of TpH.

The average resolution of the resulting map was assessed to be 4.2 Å according to the 0.143 Fourier shell correlation (FSC) criterion (Rosenthal & Henderson, 2003 ▸; see Fig. S2). The density volume showed the expected features for the region of the cylinder wall, however the single FUs of the inner collar (ten copies of Fu-d* and ten copies of Fu-g) were again not resolved [Fig. 1 ▸(*c*)]. Interestingly, the inner collar showed the same overall architecture as shown in the previous X-ray structure of TpH and low-resolution negative-stain reconstruction of type 4 hemocyanins.

In the case where the inner collar shows *C*_5_ symmetry, it is expected to be shifted towards one of the peripheral tiers of the cylinder wall, similarly to type 1 and type 3 hemocyanin [Figs. S1(*a*) and S1(*c*)]. Misalignment of the particles due to the overall *D*_5_ symmetry of the wall might be the reason for the ‘artificial’ *D*_5_ symmetry of the inner collar obtained after 3D refinement with *C*_5_ symmetry imposed. To further clarify this, we first aimed to obtain a more reliable initial model. We performed a second round of 2D classification with a reduced number of members per group in order to obtain a larger number of more precise 2D class averages. We then used them as the input to calculate initial models both with *C*_5_ and no symmetry imposed using the validation of individual parameter reproducibility (VIPER) approach.

Surprisingly, VIPER with *C*_5_ symmetry imposed produced an initial model with an artificial *D*_5_ overall symmetry and 40 densities within the inner collar (instead of 20), similar to the previous X-ray structure of the complex. On the other hand, VIPER with no symmetry imposed revealed a striking overall architecture with a typical *D*_5_ symmetrical wall and 20 FUs inside organized in a complex asymmetric manner [Fig. 1 ▸(*d*)].

### 2.2. Refinement of particles including a symmetry mismatch   

To further understand this initial structure, we used the asymmetric VIPER volume as the reference for a high-resolution asymmetric refinement using *MERIDIEN* (Moriya *et al.*, 2017 ▸) against the particles extracted from the selected ISAC class averages. However, we were not able to refine the structure using the standard approach, probably because the local *D*_5_ symmetry of the cylinder wall of the reference volume (75% of the particle density) introduced multiple local minima during the primary angle search and a high degree of ‘smearing’, although we did not impose any symmetry during the refinement.

Symmetry mismatches have been noted for several macromolecular complexes. Examples are, among others, the ABC toxin complexes (Gatsogiannis *et al.*, 2018 ▸; Meusch *et al.*, 2014 ▸), the ClpXP and ClpAP proteases (Baker & Sauer, 2012 ▸; Beuron *et al.*, 1998 ▸), the 26S proteasome (de la Peña *et al.*, 2018 ▸), and several phages (Koning *et al.*, 2016 ▸). Structure determination of symmetry mismatched and/or pseudo-symmetric complexes by cryo-EM and single-particle analysis is challenging and several computational approaches have been therefore proposed to tackle their structural analysis at near atomic resolution including localized reconstruction and signal subtraction (Bai *et al.*, 2015 ▸; Ilca *et al.*, 2015 ▸), local volume symmetrization (Sindelar & Downing, 2007 ▸), extensive 3D sorting (Roh *et al.*, 2017 ▸), and data-set symmetrization (Quentin *et al.*, 2018 ▸).

Here we applied a simple local reference-symmetrization approach during the asymmetric structure refinement (Fig. S3) (Gatsogiannis *et al.*, 2018 ▸), *i.e.* after each refinement round, the density of the wall region was symmetrized using *D*_5_ symmetry, whereas the density of the inner collar was scaled in order to put an additional weight on this region during the refinement. Finally, both densities (*D*_5_ cylindrical wall and *C*_1_ weighted inner collar) were combined into one, which was used as the reference for the subsequent refinement iteration. These steps were performed using the ‘user function’ capability of *MERIDIEN* (a custom Python script with a sequence of operations for adjustment of the reference volume after each iteration). A template ‘user function’ can be downloaded from http://sphire.mpg.de and can be easily modified and expanded to handle, in a similar manner, volumes including a symmetry mismatch or even multiple symmetry mismatches.

This strategy was applied during the first refinement rounds of the asymmetric refinement in order to obtain global projection parameters. Afterwards, we performed local refinements without any adjustment of the reference volume and deter­mined the structure of the complex at an overall resolution of 5.1 Å [Figs. 1 ▸(*e*), S3 and S4]. Thus, with the help of reference adjustment during the first iteration rounds, we were able to solve the structure of TpH after a single asymmetric refinement run. Furthermore, the two structural components were not treated independently during processing, allowing the analysis of the interfaces at the symmetry mismatch.

### 2.3. Cryo-EM structure of TpH   

**Figure 2:**
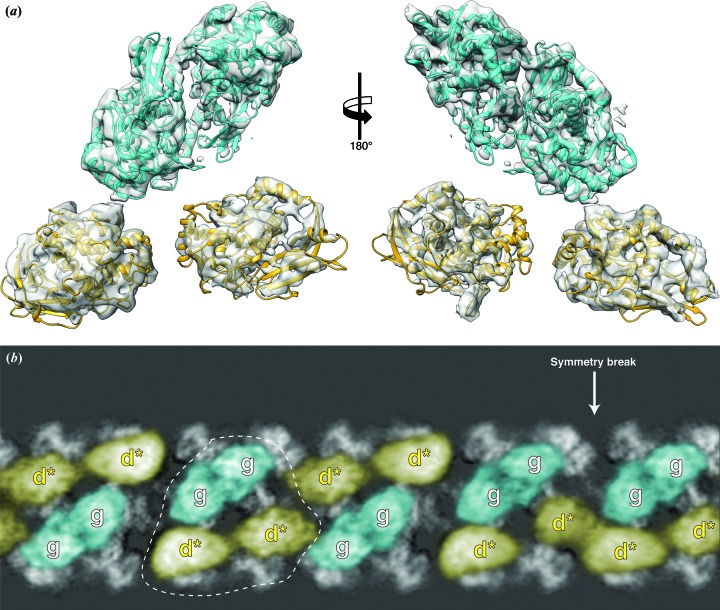
Asymmetric architecture of the inner collar. (*a*) Extracted cryo-EM density of a set of inner FUs with the respective homology models fitted. (*b*) The cryo-EM structure depicted in cylindrical sections. FU-gs, and FU-d*s are shown in cyan and yellow, respectively. The wall region is shown in gray. The arrow indicates the area of the symmetry break. The dashed line indicates a set of inner FUs, *i.e.* two FU-gs and two FU-d*s

**Figure 3:**
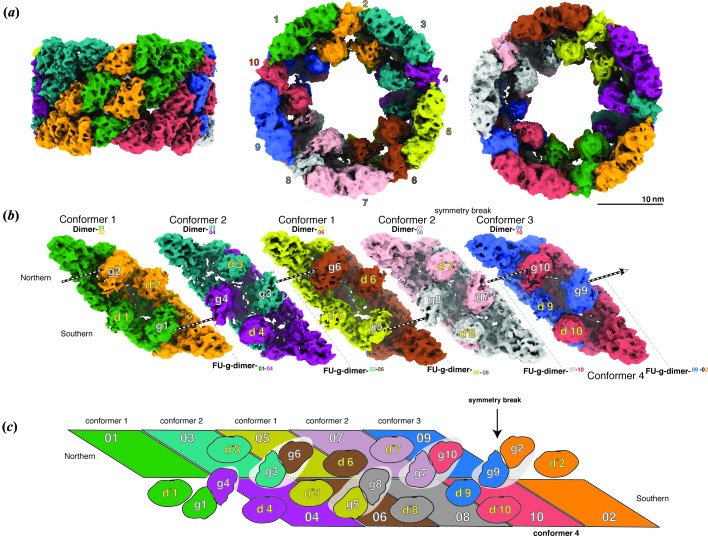
Architecture of TpH. (*a*) Cryo-EM structure of TpH decamer in side, top and bottom view. The ten protomers are highlighted in color. (*b*) Five protomer-dimers viewed from inside the cylinder. Colors correspond to those of (*a*). FUs of the inner collar (g and d*) are indicated. FU-gs forming FU-g dimers are indicated. (*c*) Schematic of the decameric assembly shown with the cylinder unrolled and shown from inside. FU-g dimers are highlighted by the surrounding white background. Colors of each protomer correspond to those of (*a*) and (*b*). The arrow indicates the region of the symmetry break.

The revealed structure shows a typical wall of hemocyanin with *D*_5_ symmetry and a characteristic asymmetric architecture of the inner-collar domains. The resolution of the inner-collar domains was lower in comparison with the wall domains and not sufficient for *de novo* molecular modeling [Fig. 1 ▸(*e*)]. Therefore, we constructed the entire structure model by rigid-body fitting. First, we fitted the available crystal structure of the entire wall (60 FUs) into our density volume. Both structures showed an excellent agreement and further adjustment of the model by flexible fitting was not necessary. Subsequently, the inner domains were constructed by superposing homology models of FU-g and FU-d* into each respective density [Fig. 2 ▸(*a*)]. The densities of the linker peptides are not resolved in the final map. We have however, considered the distance between the N- and C-termini of FU-d to FU-d*, FU-d* to FU-e and FU-f to FU-g, and the length of the respective linker peptide (Table S1 of the supporting information), and finally determined the pathways of the FUs of all ten subunits unambiguously (Fig. 3 ▸).

In all known molluscan hemocyanins described so far, ten copies of FU-g form dimers that arrange in a circular manner to form an inner collar with *C*_5_ symmetry that is shifted towards one cylinder opening (northern hemisphere of the lumen) (Fig. S1). In TpH, the FU-gs form typical FU dimers, but in this case the dimers unusually occupy the northern and southern hemispheres of the inner space alternately [cyan FUs in Figs. 1 ▸(*e*) and 2 ▸(*b*)]. The duplicated FU-d*s do not form FU dimers. They are located instead in the void space opposite the typical FU-g dimers [yellow FUs in Figs. 1 ▸(*e*) and 2 ▸(*b*)]. Furthermore, because of the north and south alternate orientation of the five FU-g dimers, the first and last dimers are necessarily located on the northern side next to each other, breaking the symmetry [Fig. 2 ▸(*b*)].

Hereafter, beginning with the first protomer of the northern hemisphere after the symmetry break [green protomer in Fig. 3 ▸(*a*)] in a clockwise direction we designate the protomers as protomer 01 to protomer 10. Throughout the manuscript we also use the following nomenclature to describe the arrangement of the domains: FU-g-01 (FU-g of protomer 01) and FU-g-04 (FU-g of protomer 04) form an FU-g dimer on the southern side of the lumen. This dimer hereafter will be referred to as FU-g-dimer-_01–04_, in which protomer names 01–04 are shown as subscript if the dimer is located on the southern side, and superscript if located on the northern side [Figs. 3 ▸(*b*) and 3(*c*)].

### 2.4. The TpH subunit adopts four different conformations   

**Figure 4:**
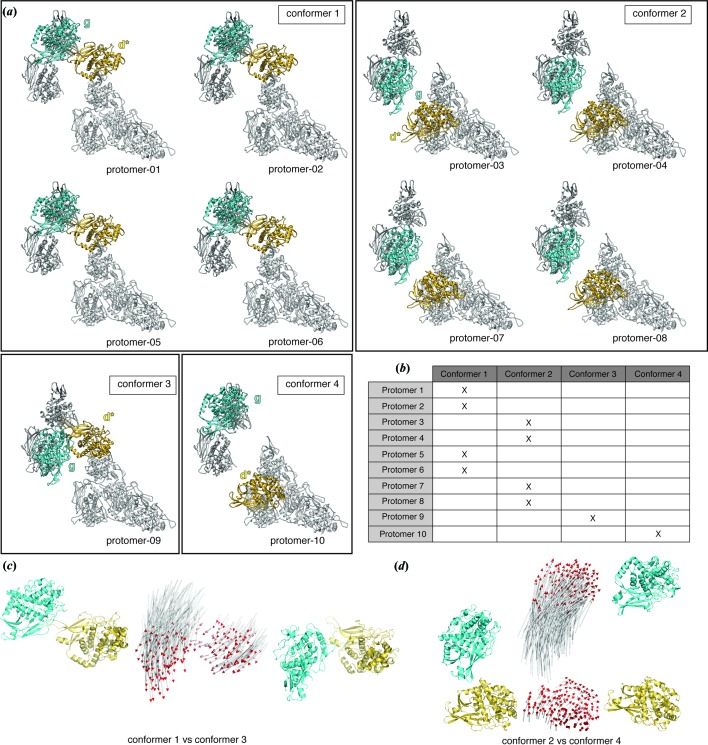
Subunits of TpH acquire four different conformations. (*a*) The ten protomers of TpH can be assigned to four conformers based on the position and orientation of FU-d*s and FU-gs. Molecular models are shown as ribbon diagrams. FU-d* and FU-g are shown in yellow and cyan, respectively. The wall region is colored gray. (*b*) Classification of the ten protomers in four conformers (*c* and *d*) Comparison of FU-d*s and FU-gs between conformer 1 and 3 (*c*) and between conformer 2 and 4 (*d*). FU-d* (yellow) and FU-g (cyan) of conformer 1 (left) and 3 (right), after aligning the wall regions, are shown (*c*). Vectors in the middle indicate the direction of motion between the collar FUs from conformer 1 to conformer 3. Those between conformer 2 (left) and 4 (right) are shown in (*d*).

The segment of the wall FUs (a-b-c-d,e-f) is invariant for all ten protomers and there are two different possible conformations for each FU-g and FU-d* within a single protomer, thereby resulting in four different protomer types, namely conformers 1, 2, 3 and 4 (Movie S1 of the supporting information). Thus, the four conformers possess the same primary sequence but differ in the position of their FU-d*s and FU-gs [Fig. 4 ▸(*a*)].

The decamer contains four copies of conformer 1 (protomers 01, 02, 05 and 06), four copies of conformer 2 (protomers 03, 04, 07 and 08), and a single copy of each conformer 3 and 4 (conformer 3: protomer 09, conformer 4: protomer 10) [Fig. 4 ▸(*b*)].

Conformer 1 and 2 subunits form homodimers exclusively, whereas conformers 3 and 4 form a heterodimer (dimer-^09^_10_), which is located at the region of the symmetry break. The final assembly consists of four twofold symmetrical protomer-dimers and one asymmetric protomer-dimer [Figs. 3 ▸(*b*) and 5 ▸(*a*)].

In all homodimers, protomers assemble with twofold symmetry, whereas in the heterodimer, FU-g and FU-d* are asymmetrically arranged and twofold symmetry is maintained only in the wall. The homodimers are alternately arranged in a 1–2–1–2 manner, and finally a fifth heterodimer of conformers 3 and 4 (dimer-^09^_10_) closes the wall (Figs. 3 ▸ and S5), thereby breaking the twofold symmetry of the collar. It is important to note that dimer-^01^_02_ and dimer-^05^_06_ are homodimers of conformer 1, whereas dimer-^03^_04_ and dimer-^07^_08_ are homodimers of conformer 2. This assembly generates a *D*_5_ outer cylindrical wall surrounding a complex asymmetric inner structure, which is distinctive of type 4 hemocyanin.

FU-g occupies a similar position in conformers 1 and 4, as well as in conformers 2 and 3. On the other hand, the topology of the FU-d* is similar in conformers 1 and 3 (RMSD 15.3 Å), and also in conformers 2 and 4 (RMSD 6.9 Å) [Fig. 4 ▸(*a*)]. It should be noted that the orientation of FU-d* of conformers 3 and 4 is slightly different from that of conformers 1 and 2, respectively [Figs. 4 ▸(*c*) and 4(*d*)].

### 2.5. FU-g dimers plug adjacent subunit dimers   

FUs usually associate in an antiparallel manner to form twofold symmetrical FU dimers (Cuff *et al.*, 1998 ▸; Gatsogiannis *et al.*, 2007 ▸; Gatsogiannis & Markl, 2009 ▸). The FU-g dimers of *T. pacificus* also follow this pattern and are arranged with a perfect twofold symmetry, similarly to the wall FUs. The FU-g dimers are most likely to be crucial for the overall stability of the cylindrical decamer because they bridge protomers that do not form direct interactions with each other through the wall region, and therefore reinforce the inter-dimer contact zones.

The FU-g dimers are located either in the northern or southern hemisphere and connect adjacent protomer dimers [Figs. 3 ▸(*c*) and S5]. Depending on their topology (northern or southern), the FU-g dimers are rotated relative to each other by 180° along the *x* axis. Within each FU-g dimer, the two FUs are arranged antiparallel, with one FU in an orientation closer to the pole and the other FU closer to the equator (Fig. S6). FU-d*s do not form dimers, instead single copies fill void spaces between the FU-g dimers.

**Figure 5:**
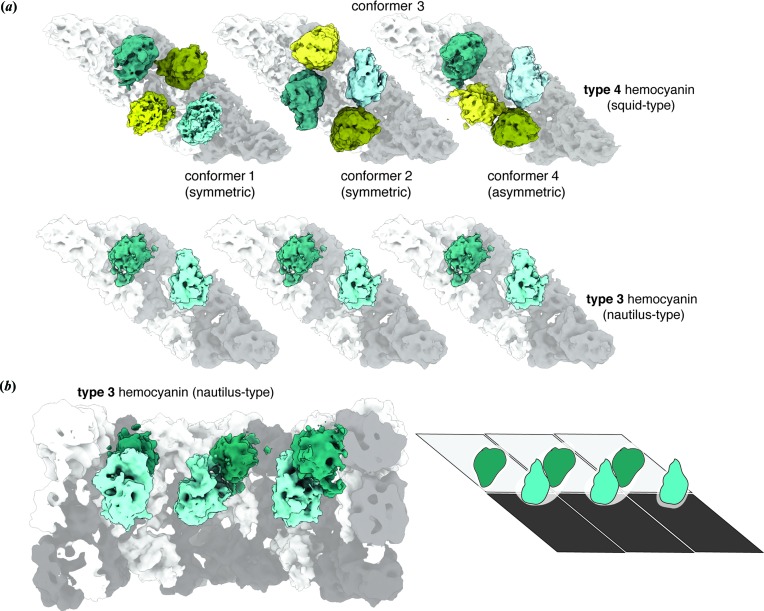
Structural comparison of TpH (type 4) with type 3 hemocyanin. (*a*) Three different dimer types that assemble the TpH decamer (type 4). The dimer of *Nautilus* hemocyanin (type 3) (EMD-1434) is also shown. Inner FUs of each subunit dimer are highlighted in color. Polar and equatorial FU-gs are colored green and cyan, respectively. FU-d*s are shown in yellow. (*b*) Cut-away view of the cryo-EM density of *Nautilus* hemocyanin (type 3). Polar and equatorial FU-gs are colored green and cyan, respectively. The wall region is shown in transparent gray. For better clarity, a schematic of the view and the density of an extracted subunit dimer are also shown.

Conformer-1 dimers supply northern- and southern-polar FU-gs on the left and right, respectively, between which two copies of FU-d* are entrapped [Fig. 5 ▸(*a*)]. Conformer-2 dimers supply southern- and northern-equatorial FU-gs on the left and right, respectively, wherein two FU-d*s are located in the void northern and southern polar spaces towards the open face of the cylinder, respectively [Fig. 5 ▸(*a*)].

Due to the 1–2–1–2 arrangement of the conformer-1 and -2 homodimers, the resulting termini display FU-gs at the northern area [Figs. 3 ▸(*b*) and S5]. These are then connected by heterodimer-^09^_10,_ because this is the only dimer displaying two copies of FU-g at the northern hemisphere. Therefore, at the left and right sides of the heterodimer, two northern FU-g-dimers are formed, *i.e.* FU-g-dimer-^07–10^ and FU-g-dimer-^09–02^ [Figs. 3 ▸(*b*), S5 and S6], thereby breaking the symmetry of the inner-collar structure. The FU-d* copies of dimer-^09^_10_ are consequently entrapped at the southern area of the heterodimer.

### 2.6. Comparison with other molluscan hemocyanins   

Squid hemocyanin evolved from type 3 (nautilus type) hemocyanin [Fig. S1(*e*)] by acquiring an additional FU, namely FU-d*. Unusually, this additional FU is not located at the C-terminal, but between wall FU-ds and FU-es (Mouche *et al.*, 1999 ▸). The additional FU does not however alter the architecture of the cylinder wall, which is indistinguishable in all molluscan hemocyanins described so far, but rather contributes to the collar complex by enlarging it and forcing it to adapt a more asymmetric and complex architecture. The unique architecture of the inner-collar complex is most probably linked to the unusual high cooperativity of squid hemocyanin, with 2–4× higher Hill coefficients than other types of molluscan hemocyanin (Decker *et al.*, 2007 ▸; Zielinski *et al.*, 2001 ▸).

Despite the duplication of FU-d in the N-terminal segment of the subunit, the cylindrical structure was maintained regardless of the dramatic change inside. Since hemocyanin is freely dissolved, alteration of the characteristic perforated cylindrical shape of the hemocyanin cylinder wall would most probably alter the mobility of floating hemocyanin and have a tremendous impact on the relative viscosity and osmotic pressure of the circulating hemolymph plasma. Nevertheless, modification of the inner structure of the cylinder by dismissal or duplication of FUs appears instead as a more appropriate evolutionary tool to fine-tune the respiratory plasticity, depending on the needs of the respective animal (Decker *et al.*, 2007 ▸; Markl, 2013 ▸; Thonig *et al.*, 2014 ▸).

Symmetry breaking in a more extensive manner was also observed for the inner domain of the tri-decameric mega-hemocyanin of ceri­thioid snails [Fig. S1(*b*)] (Gatsogiannis *et al.*, 2015 ▸). The inner cavity of the central cylindrical decamer is fully packed by ten copies of FUs f1, f2, f3, f4, f5 and f6 which are acquired by gene duplication during evolution (Lieb *et al.*, 2010 ▸). These segments of five subunit dimers acquire five different conformations to form a pyramid-like inner structure that completely fills the cylinder. The central mega-hemocyanin thus acquired 40 additional oxygen-binding sites and the ability to stack with two peripheral standard decamers to form stable tri-decamers, resulting in a more efficient oxygen transporter with exceptional respiratory plasticity. In contrast, despite the additional FU (FU-d*), squid-type hemocyanin is not able to form multi-decamers.

With regard to the inner-collar architecture, it should be emphasized that the archetypal type 3 decameric hemocyanins (nautilus-type) show a *C*_5_ symmetrical inner-collar structure, formed by a circular arrangement of five copies of FU-g dimers. In contrast to TpH, the FU-g dimers are exclusively located at the northern hemisphere [Fig. 5 ▸(*b*)] (Gatsogiannis *et al.*, 2007 ▸). In addition, type 1 molluscan hemocyanins (keyhole limpet type) with an additional terminal FU (FU-h), show a substantially enlarged inner-collar structure [Fig. S1(*a*)]. The additional ten copies of FU-h are positioned further north than the FU-g collar and cap one face of the cylinder. In this case, the decamer retains the overall *C*_5_ symmetry (Gatsogiannis & Markl, 2009 ▸; Zhang *et al.*, 2013 ▸).

The asymmetric subunit dimer-^09^_10_ of TpH (conformer 3–4) resembles the typical subunit dimers of type 1 *C*_5_ hemocyanin except for the presence of the additional FU-d*s [Fig. 5 ▸(*a*)]. In particular, this subunit dimer displays two copies of FU-gs at identical sites to type 3 hemocyanin, although it entraps an additional two copies of FU-d* at the southern area [Fig. 5 ▸(*a*)]. On the other hand, the symmetric conformer-1 dimer of TpH is composed of a pair of subunits, both possessing FU-g at the same site as the polar-FU-g of type 3 hemocyanin [Fig. 5 ▸(*a*)]. Similarly, the conformer-2 dimer is also a homodimer with both subunits possessing FU-g at the same site as equatorial-FU-g of type 3 hemocyanin [Fig. 5 ▸(*a*)].

The exclusive formation of archetypal type-3-like asymmetric subunit dimers by squids is possibly impeded by steric clashes at contacts between the two copies of the additional FU-d* and/or between FU-d* and FU-g, which occur when adjacent subunits and subunit dimers assemble. The archetypal-like subunits therefore favor the formation of homo-subunit dimers by themselves instead, *i.e.* the conformer-1 and conformer-2 homodimers, respectively [Fig. 5 ▸(*a*)]. On each homodimer, the FU-d* probably occupies a thermodynamically appropriate position, *e.g.* the equatorial FU-d* site of conformer 1 and the polar FU-d* site of conformer 2 [Fig. 5 ▸(*a*)]. The FUs are connected by long-linker peptides and are able to wobble around them when not assembled (Spinozzi *et al.*, 2012 ▸). Because of this flexibility, the FU-d*s are able to occupy two thermodynamically favored positions (equatorial and polar). However, due to steric clashes, these FU-d* sites cannot be occupied simultaneously (Fig. S7). The circular assembly of the dimers to form the cylindrical decamer is triggered by the conserved formation of FU-g dimers connecting adjacent subunit homodimers.

### 2.7. Model for the structural evolution of squid hemocyanin   

**Figure 6:**
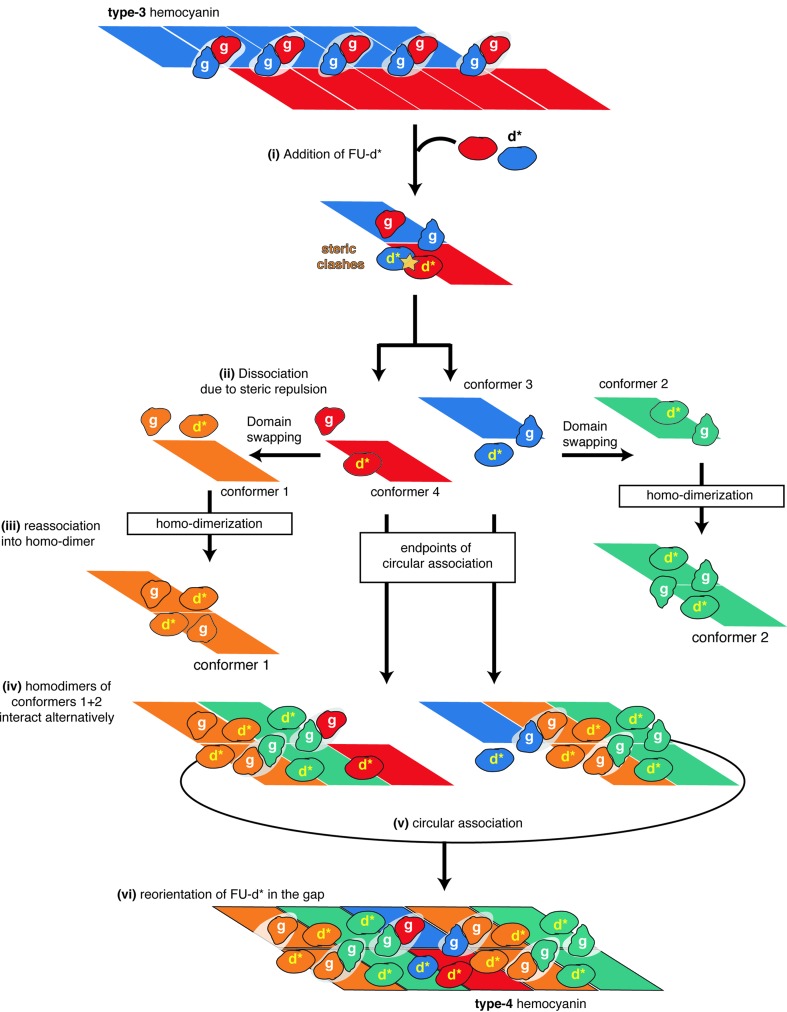
Model for the evolution from type 3 to type 4 hemocyanin. Step (i) type 3 hemocyanin acquired FU-d* by gene duplication during evolution. FU-d* are entrapped on the sites that are thermodynamically stable, which correspond to FU-d* sites of conformer 1 and 2. Step (ii) Due to steric repulsion between FU-d*s, protomer dimers favor dissociation to monomers. Step (iii) Each dissociated monomer reassociates to form a homodimer, wherein domain swapping occurrs to avoid steric repulsion. Step (iv): Homodimers assemble circularly. Step (v) The dissociated monomers bind between the terminal subunits to close the circular association. Step (vi) FU-d*s rearrange to avoid steric repulsions and a hetero-protomer dimer is formed, which closes the circle.

Considering these structural characteristics together, we propose the following scenario to explain how the asymmetric squid hemocyanin (type 3) evolved from the *C*_5_-symmetric type 1 (nautilus-type) hemocyanin (Fig. 6 ▸). The squid-type hemocyanin subunit dimer adopts a type-3-like conformation with FUs of the inner collar positioned at thermodynamically stable sites during the assembly [Fig. 6 ▸, step (i)]. However, due to steric hindrances between FU-d*s (Fig. S7), this archetypal-like subunit dimer dissociates into two protomers, in which one subunit displays a polar and the other an equatorial FU-g [Fig. 6 ▸, step (ii)]. Then, each dissociated subunit spontaneously reassociates to form homodimers, generating conformer-1 and conformer-2 dimers, respectively [Fig. 6 ▸, step (iii)]. Importantly, to avoid clashes during the dimer formation with the FU-d*s derived from the counterpart subunit, FU-d* within each subunit rearranges and occupies a thermodynamically stable position in the same hemisphere with the FU-g [domain swapping, Fig. 6 ▸, step (ii)]. This rearrangement is possible due to the flexible-linker peptides connecting subsequent FUs, allowing the FU-d*s to oscillate around them when not assembled. The two homodimers generated associate alternately [Fig. 6 ▸, step (iv)]. However, the termini do not associate because both ends have FU-g components in the northern hemisphere. To close the circular association, the dissociated type-1-like subunits come into the gap [Fig. 6 ▸, step (iv)]. To avoid steric repulsion, the FU-d*s locally rearrange, which generates conformer-3 and -4 heterodimers [Fig. 6 ▸, step (iv)].

### 2.8. Crystal structure of TpH displays artificial *D*_5_ symmetry   

Our data set contains mostly side-views, with the highly symmetric cylinder wall (75% of the density) covering the fivefold pseudo-symmetric interior. Despite this, 2D clustering using ISAC (Yang *et al.*, 2012 ▸) separated the different views of the cylinder successfully. Based on these 2D projections with an enhanced signal-to-noise ratio, we were able to reveal the correct arrangement of the inner collar and obtain a reliable initial model using the VIPER approach (Moriya *et al.*, 2017 ▸). The final volume was obtained after asymmetric refinement, which was successful only after adjustment of the reference including symmetrization of the cylinder wall and proper weighting of the inner collar during the initial refinement rounds. The present study clearly shows that the inner collar of TpH is asymmetric, whereas the outer cylindrical wall follows *D*_5_ symmetry.

In the crystal structure of TpH reported previously (Gai *et al.*, 2015 ▸), 40 anomalous signals derived from Cu_2_O_2_ clusters arranged with *D*_5_ symmetry were observed within the cylinder, although the inner collar contains only 20 FUs. The authors concluded that the observed *D*_5_ symmetry was the result of crystal packing, with two antiparallel *C*_5_ symmetrical hemocyanins arranged face-to-face.

However, the present results allow us to conclude that in the TpH crystals, the crystal contacts occur between the *D*_5_ symmetric cylinder walls of adjacent hemocyanin molecules, which contain asymmetric inner collars (Fig. S8). The *D*_5_ symmetry observed in the crystal structure of the inner collar was therefore the result of intermingling of ten different orientations between the interacting hemocyanin decamers during crystal packing. Similarly, previous low-resolution reconstructions of type-4 hemocyanins displaying overall *D*_5_ symmetry were the result of incorrect averaging and/or symmetrization (Lambert *et al.*, 1995 ▸; Mouche *et al.*, 1999 ▸).

## 3. Conclusions   

In the present study, we reveal the complete structure of type-4 molluscan hemocyanin. This hemocyanin decamer possesses an additional FU-d* in the N-terminal segment of each subunit. Our structure revealed a striking asymmetric inner-collar architecture and a conserved symmetric cylinder wall. In the interior of the cylinder, five typical FU-g dimers are located in the northern and southern area alternately. Both the first and last FU-g dimers are located in northern area, where the symmetry is broken. The FU-d*s are entrapped in the void spaces generated by the rearrangement of the FU-g dimers. The present structure allows us to propose a possible strategy of evolution of type 4 hemocyanin to entrap the additional FU-d*. The present study resolves the uncertainties in the previous crystal structure of type 4 hemocyanin in the region of the inner collar and further underlines that cryo-EM, supported by protocols that are designed to address the structures of symmetry mismatched complexes, is the only method to tackle the structure of large asymmetric complexes displaying a symmetric outer surface.

## 4. Experimental   

### 4.1. Sample preparation   

Hemolymph collected from living *T. pacificus* was centrifuged at 30 000g for 4 h. The supernatant was discarded and the blue precipitant was dissolved by 100 m*M* HEPES pH 7.5, 200 m*M* CaCl_2_. The purified hemocyanin was stored at 277 K until further analysis.

### 4.2. Cryo-electron microscopy   

A 4.5 µl aliquot of the purified hemocyanin with a concentration of 2.9 mg ml^−1^ was placed on a freshly glow-discharged holey carbon grid (Quantifoil R2/1). The sample was blotted for 2.2 s and plunge-frozen into liquid ethane using the Cryoplunge 3 System (Gatan). Samples were prescreened on a Tecnai G Spirit Microscope (FEI) operated at 120 kV, with a cryo-transfer holder 626 (Gatan).

The data set was collected with a TITAN KRIOS electron microscope (FEI) equipped with Cs corrector and XFEG operated at 300 kV, at a defocus range −0.8 to −2.2 µm, with the automated data-collection software EPU (FEI). Images were recorded with a FALCON II direct detector (FEI) operated in linear mode as a movie composed of 24 frames per image at a pixel size of 1.14 Å per pixel. The electron dose was 56 e Å^−2^ per image, which corresponds to 2.33 e Å^−2^ per frame.

### 4.3. Image processing   

Drift correction was performed using the program *MotionCor2* (Zheng *et al.*, 2017 ▸). Estimation of the contrast transfer function (CTF) was performed using *CTER* (Penczek *et al.*, 2014 ▸) in *SPHIRE*. Particles were automatically selected using *GAUTOMATCH* (unpublished work, http://www.mrc-lmb.cam.ac.uk/kzhang/Gautomatch/). Particles located on the carbon layer were manually discarded. A total of 359 250 particles were subjected to 2D clustering using the iterative stable alignment and clustering approach (ISAC; Yang *et al.*, 2012 ▸) of the *SPHIRE* program suite. Unstable particles were removed automatically during ISAC and the resulting class averages were visually inspected. The final ‘clean’ particle stack contained 196 315 single particles. An initial 3D model of TpH was calculated from the remaining 1270 high-quality 2D classes with the program *RVIPER* of the *SPHIRE* program suite without an imposed symmetry.

Further 3D structure determination was carried out with *SPHIRE* using a local symmetrization approach as previously reported (Gatsogiannis *et al.*, 2015 ▸; 2013 ▸; Meusch *et al.*, 2014 ▸). *MERIDIEN* was used for 3D refinement with the *RVIPER* model as a starting reference and applying a ‘user function’. The ‘user function’ is a custom python script that performs a series of user-defined operations to each half-volume after each refinement iteration. The half-volumes are automatically passed to the ‘user function script’ by *MERIDIEN* and after running the script, the respective output volumes are forwarded back to *MERIDIEN* and used as a reference for the next refinement iteration of the respective half-set.

A template user function for reference volume adjustment is available at http://www.sphire.mpg.de. Briefly, after each refinement iteration, the densities corresponding to the cylinder wall and interior of the cylinder were extracted. The density of the wall was symmetrized with *D*_5_ symmetry. The threshold of the inner collar was scaled to focus the refinement more on this area. The most optimal scaling factor (1.5×) was determined by running multiple 3D refinements with different scaling factors and finally choosing the weighting factor producing the highest average resolution for TpH. Subsequently, the two volumes were combined and masked to remove background noise. The resulting volume was then forwarded to the next iteration of 3D refinement. Final local refinements were performed without any adjustment to the reference volume. The average resolution was determined using the gold-standard FSC 0.143 criterion. The map was sharpened by applying a *b* factor of −190 Å^2^, that was estimated using the *PostRefiner* tool (*SPHIRE*). The correct hand of the reconstruction was confirmed by rigid-body fitting of the available crystal structure of the TpH cylinder wall (PDB entry 4yd9; Gai *et al.*, 2015 ▸) into the cryo-EM density. Local resolution estimation was performed using the *Local Resolution* tool (*SPHIRE*) and the volume was filtered accordingly using *3D Local Filter* (*SPHIRE*). Application of 3D variability and sorting revealed a population of incomplete decamers and enhanced flexibility within the asymmetric collar, most likely due to the limited number of interaction interfaces of the inner-collar domains. However, further 3D refinement of the intact decamers and individual clusters did not reveal any significant conformational changes or result in further improvement of the overall resolution.

#### 4.3.1. Modeling, fitting and visualization   

The available crystal structures of the inner-collar FUs (Gai *et al.*, 2015 ▸) and homology models of the inner-collar FUs were rigid-body fitted into the final density map using *UCSF Chimera* (Pettersen *et al.*, 2004 ▸) (Table S2). Fitting-based segmentation was also performed using *UCSF Chimera*. The direction of motion between conformers was visualized using the *ModeVectors* tool in *PyMol* (Schrödinger LLC). The display of the volume in cylindrical sections was performed with a tool provided by Tapu Shaikh, written in *SPIDER* (Shaikh *et al.*, 2008 ▸). For visualization, analysis and preparation of figures we used *ChimeraX* (Goddard *et al.*, 2018 ▸), *UCSF Chimera* (Pettersen *et al.*, 2004 ▸) and *PyMol* (Schrödinger LLC).

The EM structure and the molecular model of TpH have been deposited in the EM Data Bank and Protein Data Bank under accession codes EMD-4750 and 6r83, respectively.

## 5. Related literature   

The following reference is cited in the supporting information for this article: Thompson *et al.* (1994 ▸).

## Supplementary Material

PDB reference: squid hemocyanin, 6r83

Supporting tables and figures. DOI: 10.1107/S205225251900321X/pw5003sup1.pdf

Transformation between the four different conformations of the TpH protomer. DOI: 10.1107/S205225251900321X/pw5003sup2.mov
