# Supplementary material for: Cryo-EM reveals the asymmetric assembly of squid hemocyanin
Source: IUCrJ. 2019 Apr 5;6(Pt 3):426–37. doi: 10.1107/S205225251900321X (PMC6503924; doi:10.1107/S205225251900321X)
Supplement: Supplementary file 1 [file m-06-00426-sup1.pdf]

IUCrJ

Volume 6 (2019)

Supporting information for article:

CryoEM reveals the asymmetric assembly of squid hemocyanin

Yoshikazu Tanaka, Sanae Kato, Markus Stabrin, Stefan Raunser, Takashi Matsui and Christos Gatsogiannis

Figure S1 Structure of molluscan hemocyanins. (a)-(d) 3D volume in side view (left), schematic representation of the subunit (center) and side view of the 3D volume with the cylinder wall in transparent to show the structure of the inner collar (right) shown for type 1 (keyhole limpet-type) (a), type 2 (mega hemocyanin-type) (b), type 3 (nautilus-type) (c) and type 4 (squid-type) (d). The cylinder wall, FUs-g, FUs-h, FUs-f* are shown in gray, cyan, orange and red, respectively, as indicated in the schematic representation of the respective subunit. All 3D surfaces were computed at 10 Å from the available atomic models. The original cryoEM densities of type 1, type 2, type 3 are available at the EMDB (EMD-1569, 6185, 1432). A crystal structure of the cylinder wall of type 4 hemocyanin is available at PDB -4YD9. e) Phylogenic tree of molluscan hemocyanins. The phylogram was constructed with ClustalW2.1 using the neighbor-joining method based on the amino acid sequences.(Thompson et al., 1994) KLH (*Keyhole limpet* hemocyanin [DDBJ: AJ698341]) and HtH (*Haliotis tuberculate* hemocyanin [DDBJ: AJ252741]) classified into type 1, MtH (*Melanoides tuberculate* hemocyanin [DDBJ: KC405576]) into type 2, NpH (*Nautilus pompilius* hemocyanin [DDBJ: AJ619741]) and OdH (*Enteroctopus dofleini* hemocyanin [DDBJ: AY751301] into type 3, and TpH (*Todarodes pacificus* hemocyanin [DDBJ: AB897790] and SoH (*Sepia officinalis hemocyanin* [DDBJ: DQ388569]) into type 4. Bootstrap values are given at the nodes. Scale bar, 0.1 expected changes per site.

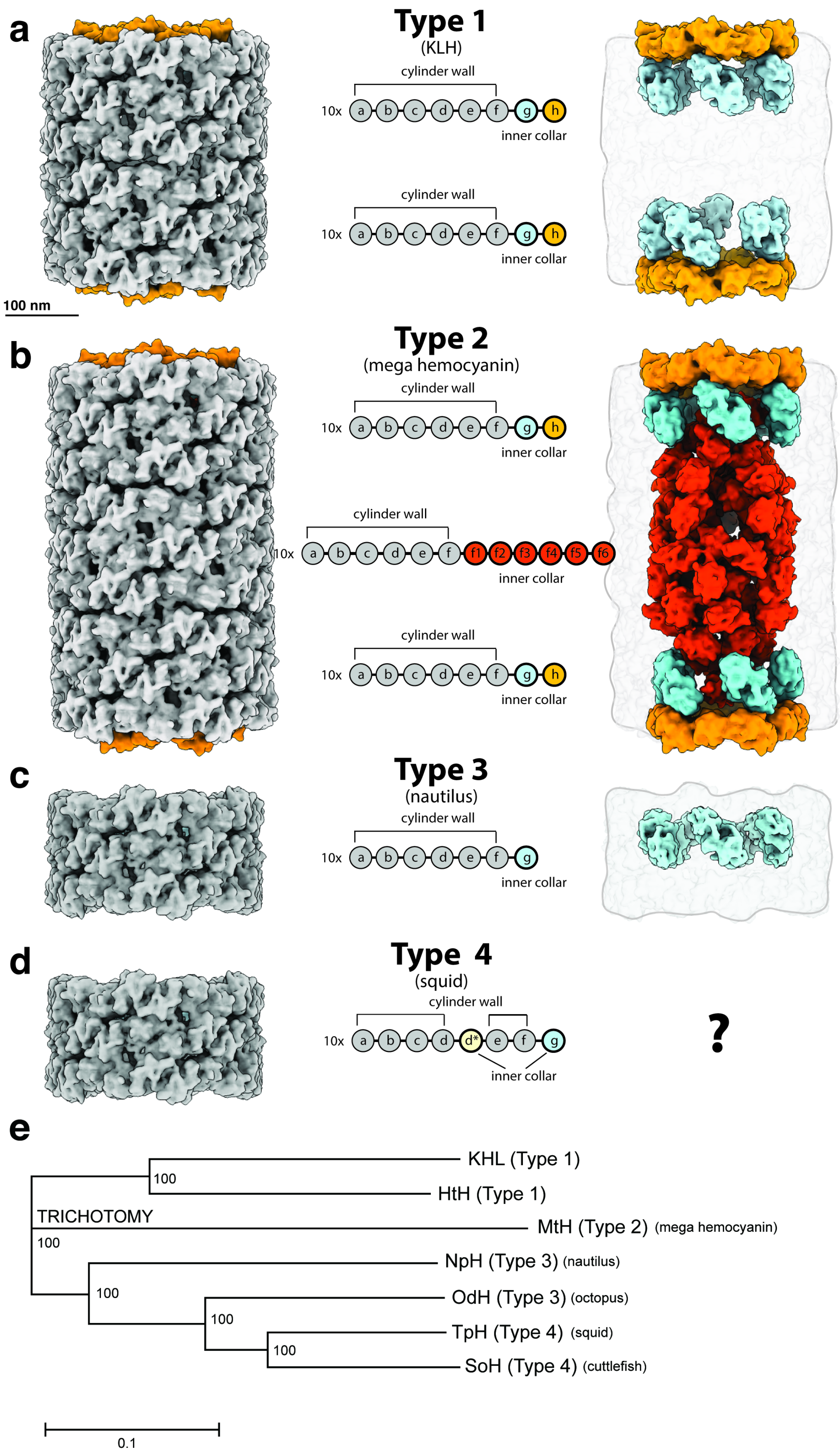
a
Type 1
(KLH)
cylinder wall
10x a b c d e f g h
inner collar
cylinder wall
10x a b c d e f g h
inner collar
100 nm
b
Type 2
(mega hemocyanin)
cylinder wall
10x a b c d e f g h
inner collar
cylinder wall
10x a b c d e f f1 f2 f3 f4 f5 f6
inner collar
cylinder wall
10x a b c d e f g h
inner collar
c
Type 3
(nautilus)
cylinder wall
10x a b c d e f g
inner collar
d
Type 4
(squid)
cylinder wall
10x a b c d d* e f g
inner collar
?
e
KHL (Type 1)
100
HtH (Type 1)
TRICHOTOMY
MtH (Type 2) (mega hemocyanin)
100
NpH (Type 3) (nautilus)
100
OdH (Type 3) (octopus)
100
TpH (Type 4) (squid)
100
SoH (Type 4) (cuttlefish)
0.1

Figure S2 CryoEM analysis of TpH with C_5 symmetry imposed

(a) Cryo-EM structure of TpH refined with C_5 symmetry imposed. Note the fragmented density of the inner collar domains. (b) Fourier shell correlation (FSC). (c) C_5 volume of TpH shown in top view, colored according to the local resolution. Note the low local resolution of the inner collar domains d) Density map of a wall FU (FU-e, protomer 1) superimposed with the molecular model. The inset shows the central α-helices of this FU.

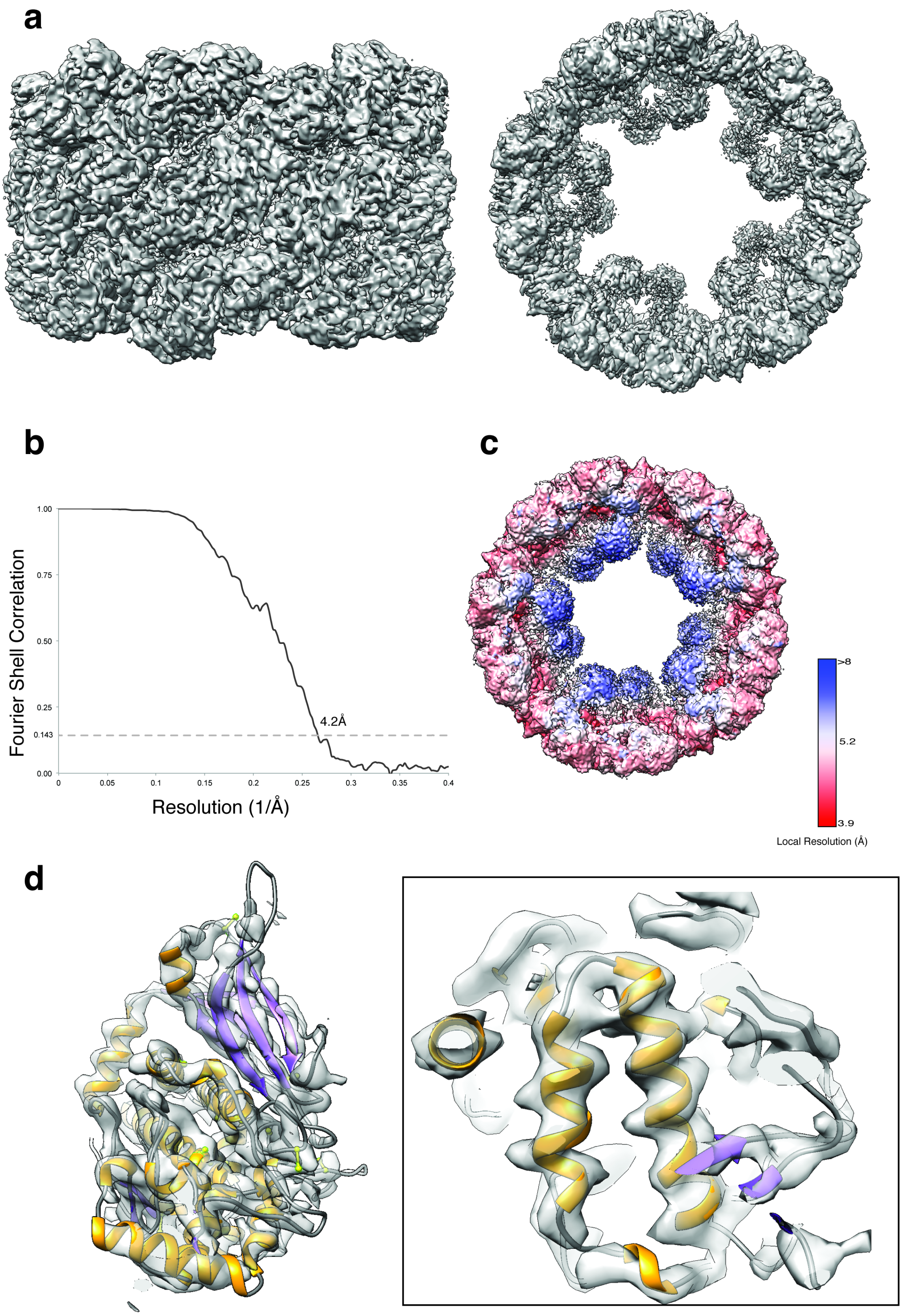
a
b
Fourier Shell Correlation
1.00
0.75
0.50
0.25
0.143
0.00
0
0.05
0.1
0.15
0.2
0.25
0.3
0.35
0.4
4.2Å
Resolution (1/Å)
c
>8
5.2
3.9
Local Resolution (Å)
d

Figure S3 Processing workflow. 2D class averages are computed using the ISAC approach(Yang et al., 2012). Selected class averages displaying an isotropic angular distribution are used to compute an initial 3D model with RVIPER. The members of these class averages are then subjected to an asymmetric 3D refinement (MERIDIEN), using the VIPER model as starting reference. After each iteration round, the resulting volume is adjusted using a custom Python script (user function) that performs a sequence of operations (inset) and subsequently forwarded as reference for the next round of refinement. This procedure is only performed during the first iteration rounds in order to obtain global projection parameters. During the local refinements, the user function is not applied.

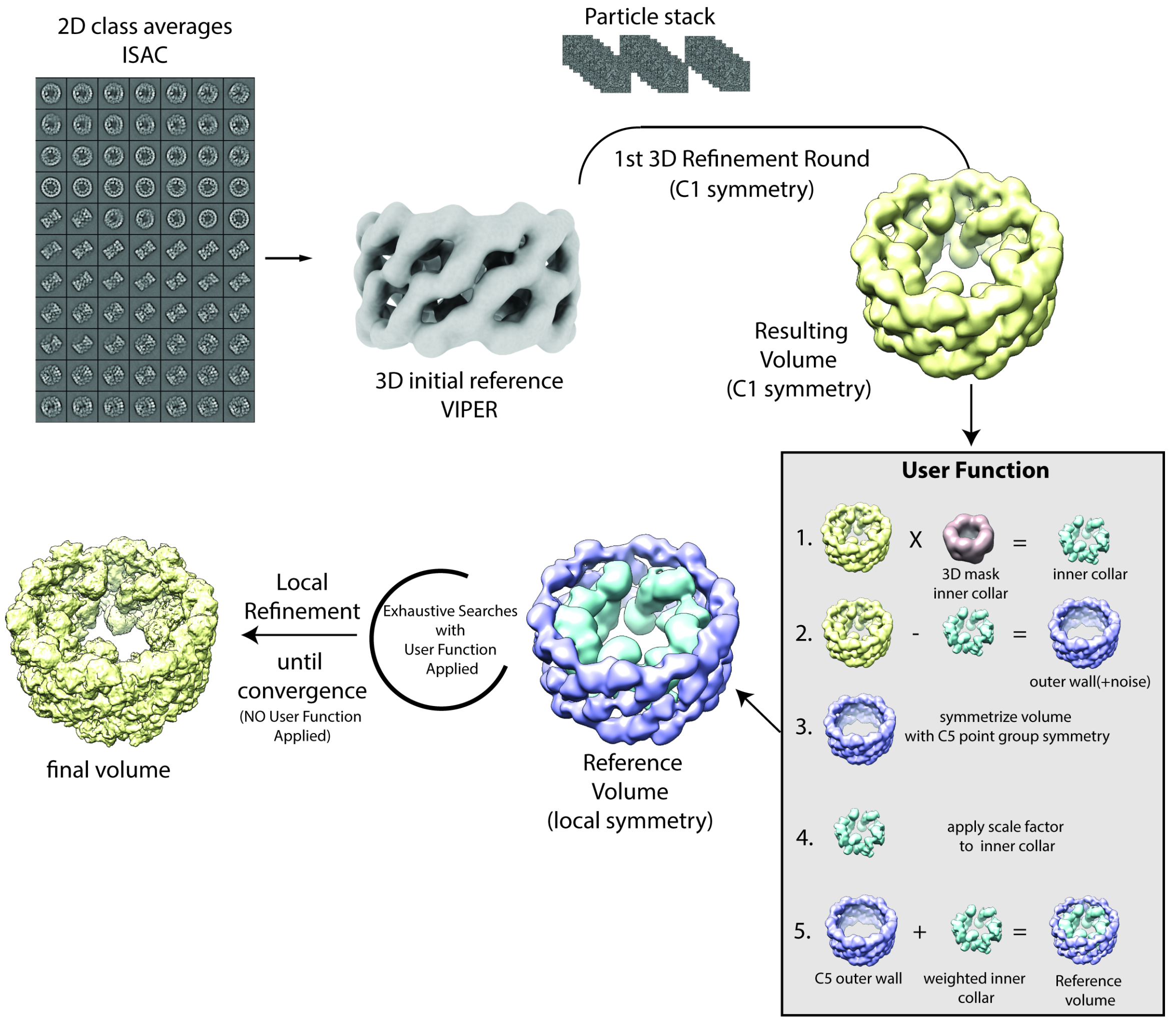

Figure S4 CryoEM analysis of TpH with no symmetry imposed

(a) Fourier shell correlation (FSC). b) Angular distribution of the particles for the final refinement round. c) Low-pass filtered density volume of TpH shown in top-, side-view and cross-section of the side view, colored according to the local resolution. (d)-(e) Representative area of the density map of a wall FU (upper image), inner collar FU-d (middle image) and inner collar FU-g (lower image) superimposed with the molecular model.

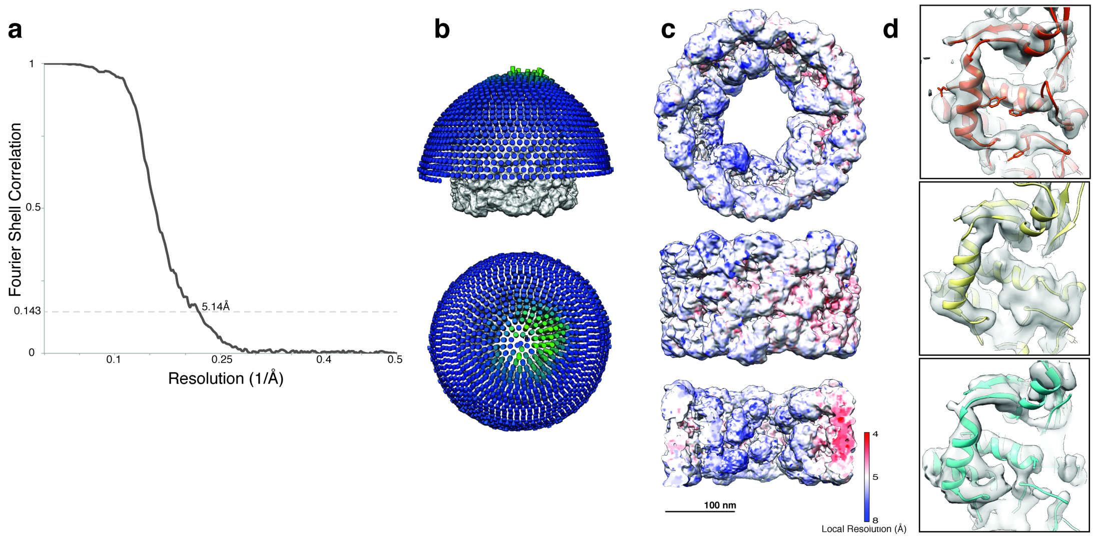

Figure S5 Schematic representation of the decameric assembly. Colors of each protomer correspond to those of Fig. 6.

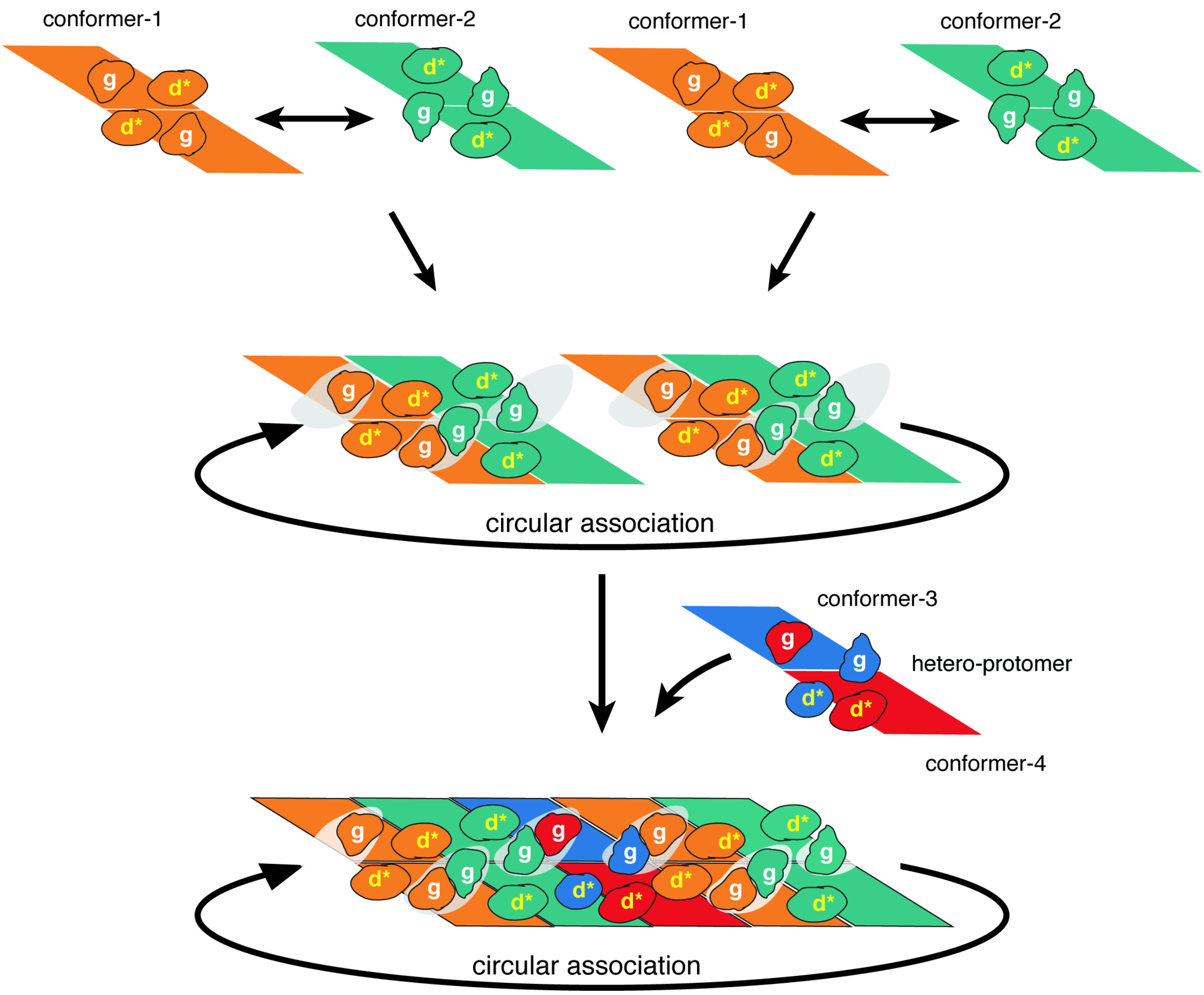

Figure S6 Southern-Northern alternate assembly of FU-g dimers. (a-e) Shown are molecular surfaces of the five FU-g dimers formed between adjacent subunit dimers. Other FUs are shown as ribbon diagrams. The dashed line indicates the interface between the adjacent subunit dimers. Colors correspond to those of Fig. 3. For better clarity, schematic representations of the FUs are also shown.

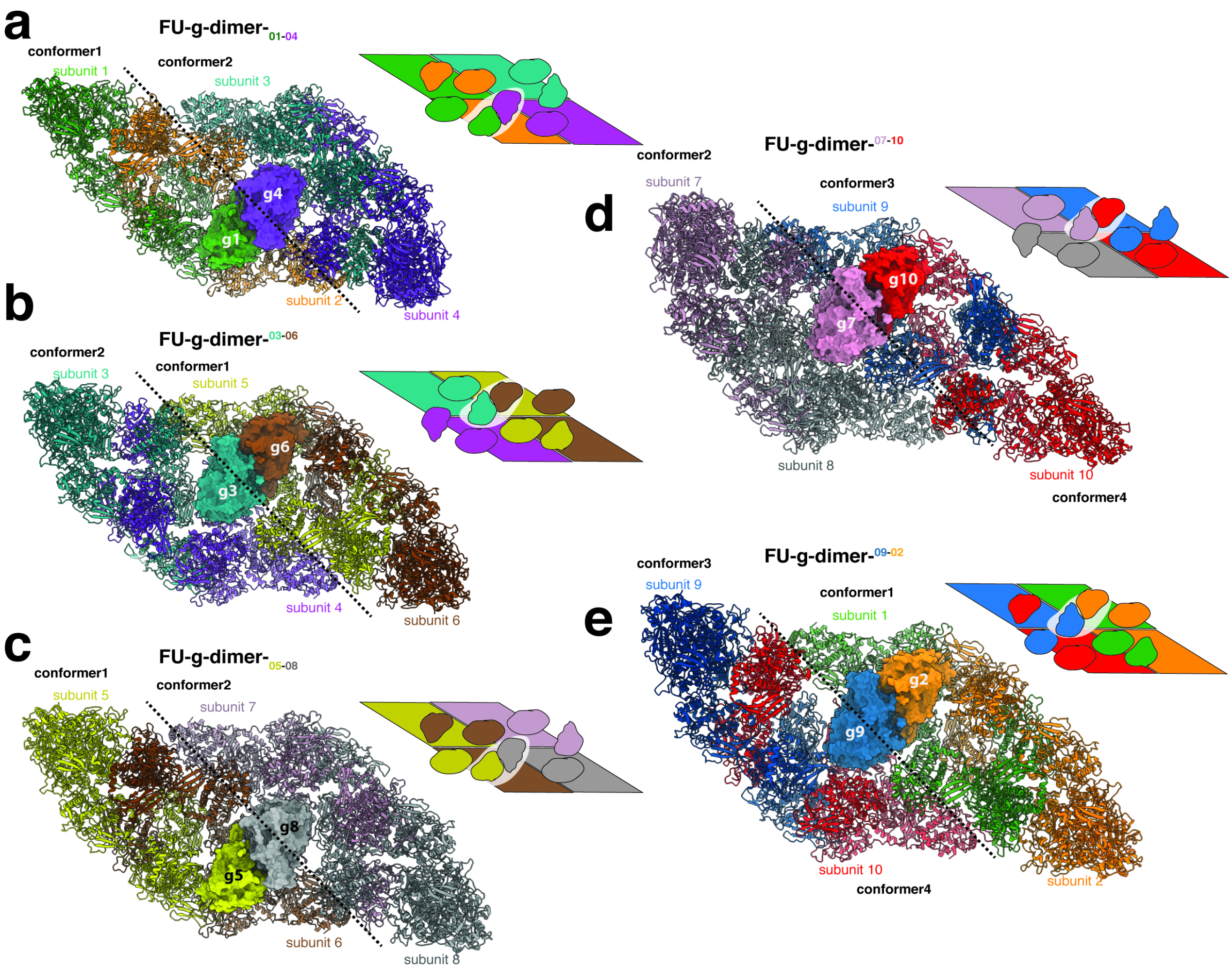

Figure S7 Putative model of protomer-dimer possessing FUs-d* on the sites corresponding to those of conformer-1 and -2 simultaneously. In the protomer-dimer, a FU-d* sterically conflicts with another FU-d* of the counterpart protomer, meaning that FU-d* sites of conformer-1 and -2 cannot be occupied simultaneously.

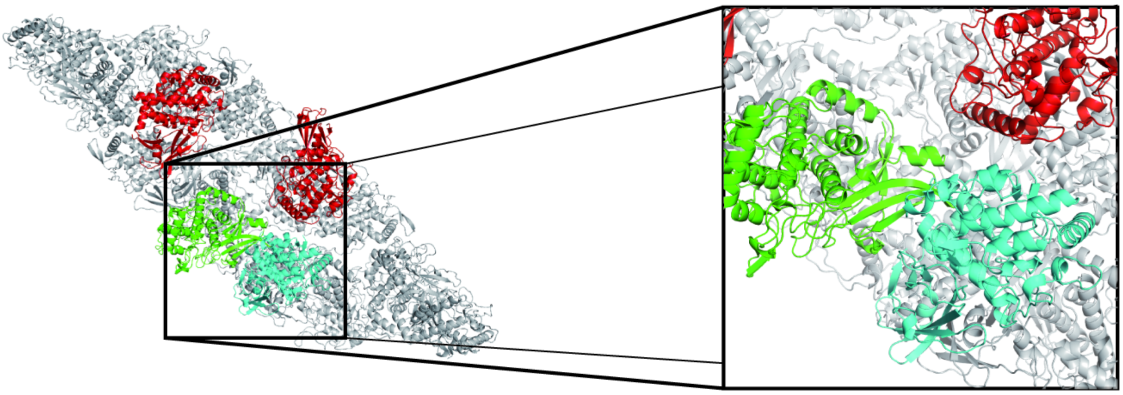

Figure S8 Crystallographic analysis of Type 4 Hemocyanin. The FUs of the outer cylinder wall of Type 4 hemocyanin are arranged with D_5 symmetry. Crystal packing involves therefore contacts between the cylinder walls of adjacent hemocyanins along their D_5 symmetry axis. The inner collar does not however follow this symmetry. Subsequent crustal structure analysis results there into incorrect averaging of 10 possible different orientations of the asymmetric inner collar. The resulting overall structure shows therefore an artificial D_5 overall symmetry and all FUs of the inner collar are consequently not resolved.

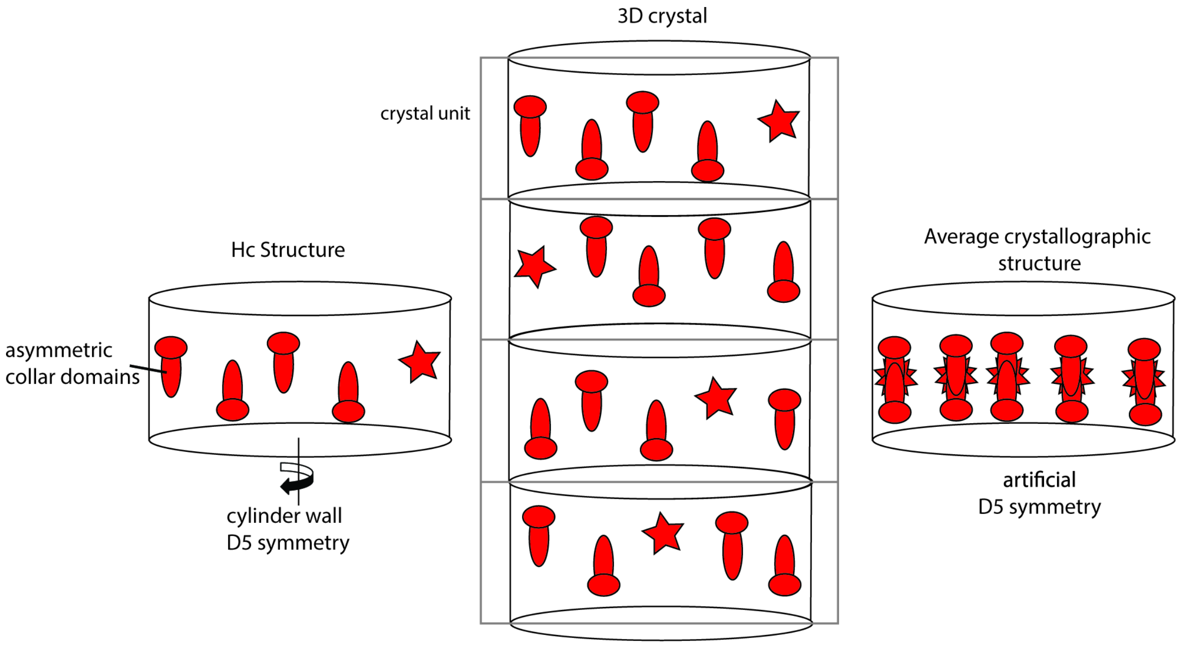

Supplementary Movie 1. Four different conformations of the TpH subunit. The video shows a morph between the four different conformations of the TpH protomer.

Table S1 Length of the linker peptides connecting FUs

FU	Domain region	Linker length (aa)
a	1-405	17
b	422-824	13
c	837-1238	19
d	1257-1654	18
d*	1672-2069	17
e	2086-2490	13
f	2503-2905	16
g	2921-3009	-

Table S2 CCFs of rigid body fitted subunits into the cryoEM density map

Protomer	correlation
1	0.8592
2	0.8581
3	0.8664
4	0.8696
5	0.8647
6	0.8561
7	0.8574
8	0.8608
9	0.8361
10	0.8496
